# Adam13 interacts with large protein complexes to regulate histone modification and gene expression

**DOI:** 10.3389/fcell.2026.1824619

**Published:** 2026-05-07

**Authors:** Ankit Pandey, Helene Cousin, Shiv Kumar, Louis Taylor, Ashmita Chander, Kelsey Coppenrath, Nikko-Ideen Shaidani, Marko Horb, Dominique Alfandari

**Affiliations:** 1 Department of Veterinary and Animal Sciences, University of Massachusetts Amherst, Amherst, MA, United States; 2 Marine Biological Laboratory, Eugene Bell Center for Regenerative Biology and Tissue Engineering and National Xenopus Resource, Woods Hole, MA, United States; 3 Human Genetics Program, Sanford Burnham Prebys Medical Discovery Institute, La Jolla, CA, United States

**Keywords:** ADAM, ARID3a, histone methylation, neural crest cell, *TFAP2A*, *Xenopus*

## Abstract

Cranial neural crest (CNC) cells are a key stem cell like tissue that contribute to most of the facial structures in vertebrates. A disintegrin and metalloproteinase (ADAM) family of proteins is essential for the induction and migration of the CNC. We have shown that Adam13 interacts with the transcription factor Arid3a to regulate gene expression; we show that Adam13 modulates histone modifications in the CNC and that Arid3a binding to the *tfap2α* promoter is dependent on the presence of Adam13. These associations promote the expression of a certain *tfap2α* variant expressed in the CNC that uniquely activates the expression of genes critical to CNC migration. Furthermore, we show that both Adam13 and human ADAM9 are associated with proteins involved in histone modifications and RNA splicing (a function critically affected by the loss of Adam13). Thus, we propose that ADAMs may act as extracellular sensors to modulate chromatin availability, leading to changes in gene expression and splicing.

## Introduction

Cranial neural crest cells (CNC) are a transient population of multi-potent cells induced during the early stages of vertebrate embryogenesis. These cells originate from the lateral edges of the neural plate and migrate extensively before differentiating into a wide array of derivatives, including most of the craniofacial skeleton and components of the peripheral nervous system (Plouhinec et al., 2017). The induction and migration of CNC cells are tightly regulated by multiple signaling pathways, including Wnt, BMP, and FGF, as well as various chemoattractants and chemorepellents that guide the cells along specific migratory paths toward their target structures (Koontz et al., 2023).

We have previously identified four members of the ADAM family as key regulators of CNC specification and migration (Alfandari et al., 2001; McCusker et al., 2009; Neuner et al., 2009; Pandey et al., 2023). Among these, Adam13 (*Xenopus* homolog of human ADAM33) plays dual roles: it proteolytically cleaves cell adhesion molecules, such as cadherin-11 and protocadherin PCNS, to release extracellular fragments that promote cell migration (Khedgikar et al., 2017; McCusker et al., 2009). In the case of cadherin-11, the cleaved fragment activates the EGFR2 receptor to stimulate AKT phosphorylation independently of its classical homophilic binding activity (Abbruzzese et al., 2016; Mathavan et al., 2017). In the neural crest, ADAMs have been implicated in modulating cell adhesion and epithelial-to-mesenchymal transition (EMT) by cleaving cadherins, including E-cadherin and cadherin-6B (Maretzky et al., 2005; Reiss et al., 2005; Schiffmacher et al., 2014).

In addition to their proteolytic functions, Adam proteins exhibit critical non-proteolytic roles; for instance, Adam11 is a non-catalytic protein that has been shown to negatively regulate Wnt signaling and positively regulate BMP4 signaling to control CNC proliferation and EMT (Pandey et al., 2023). Notably, we previously demonstrated that Adam13 undergoes self-cleavage within its cysteine-rich domain (Gaultier et al., 2002) to become a substrate for γ-secretase-mediated intramembrane cleavage (Cousin et al., 2011). The cytoplasmic domain released subsequently translocates to the nucleus and regulates the expressions of numerous genes, including *Tfap2α*, *PCNS*, and *Calpain8*, which are all essential for CNC migration (Cousin et al., 2011).

Although nuclear localization has been observed for some ADAMs and matrix metalloproteinases (MMPs), their precise nuclear functions remain largely unknown (Endsley et al., 2014; Gouignard et al., 2023). For example, ADAM9 was found to be associated with chromatin in esophageal squamous cell carcinoma under hypoxic conditions, and chromatin immunoprecipitation (ChIP) assays have revealed its associations with the promoters of hypoxia-repressed genes (Lin et al., 2021).

ARID3a (also known as Dril1 or BRIGHT) is a transcription factor involved in early decisions on cell fates in mammals (Rhee et al., 2014); it plays a crucial role in mesoderm induction and gastrulation in *Xenopus* by acting downstream of the Smad proteins to regulate TGFβ signaling (Callery et al., 2005). We previously showed that Adam13 physically interacts with Arid3a to enhance the expression of *Tfap2α*, which is a key transcription factor required for CNC induction and maintenance (Khedgikar et al., 2017; Luo et al., 2003; Rothstein and Simoes-Costa, 2020). Loss of *Tfap2α* has been reported to cause craniofacial defects across all vertebrate models studied (Barrallo-Gimeno et al., 2004; Erickson et al., 2018; Green et al., 2015; Luo et al., 2003; Milunsky et al., 2008). In mice, *Tfap2α* knockout leads to perinatal lethality with severe craniofacial and neural tube defects (Schorle et al., 1996). In humans, *TFAP2A* mutations cause branchiooculofacial syndrome, underscoring the evolutionary conservation of its function (Milunsky et al., 2008).

Arid3a functions as both an activator and a repressor of gene expression (Rhee et al., 2014). In *Xenopus*, Arid3a has been shown to activate *lhx1* expression during nephric tubule regeneration by modulating the chromatin landscape at its promoter through interaction with Kdm4 to remove the repressive H3K9me2/3 histone marks (Suzuki et al., 2019). Thus, Arid3a regulates transcription through Smad cofactors during mesoderm induction and epigenetic mechanisms during organogenesis. However, in the neural crest, the Adam13–Arid3a interaction does not appear to involve Smad proteins (Khedgikar et al., 2017), suggesting that it may instead modulate gene expression via chromatin remodeling.

Recent studies have demonstrated that dynamic chromatin remodeling is essential for CNC induction and migration (Azambuja and Simoes-Costa, 2021; Hovland et al., 2022; Linares-Saldana et al., 2021; Merkuri et al., 2024). In parallel, gene regulatory networks (GRNs) governing CNC development have been mapped extensively by identifying key transcription factors, such as SOX9, FOXD3, SNAI1/2, and the TFAP2 family (Martik and Bronner, 2021). Moreover, alternative splicing (AS) has emerged as an important layer of CNC regulation, involving several craniofacial birth defects in humans linked to mutations in the splicing regulators (Bain et al., 2016; Beauchamp et al., 2020; Berube-Simard and Pilon, 2019; Griffin and Saint-Jeannet, 2020; Loiselle and Sutherland, 2018; Marques et al., 2016).

In the present study, we show that Adam13 regulates *Tfap2α* isoform expression by modulating Arid3a activity; this involves promoting its binding to one *Tfap2α* transcription start while repressing another. Furthermore, we demonstrate that Adam13 plays a broader role in regulating histone modifications both globally and at the *Tfap2α* promoter in CNC cells, revealing a previously unappreciated epigenetic function for this metalloproteinase.

## Results

### Loss of Adam13 in the CNC affects both gene expression and splicing

**FIGURE 1:**
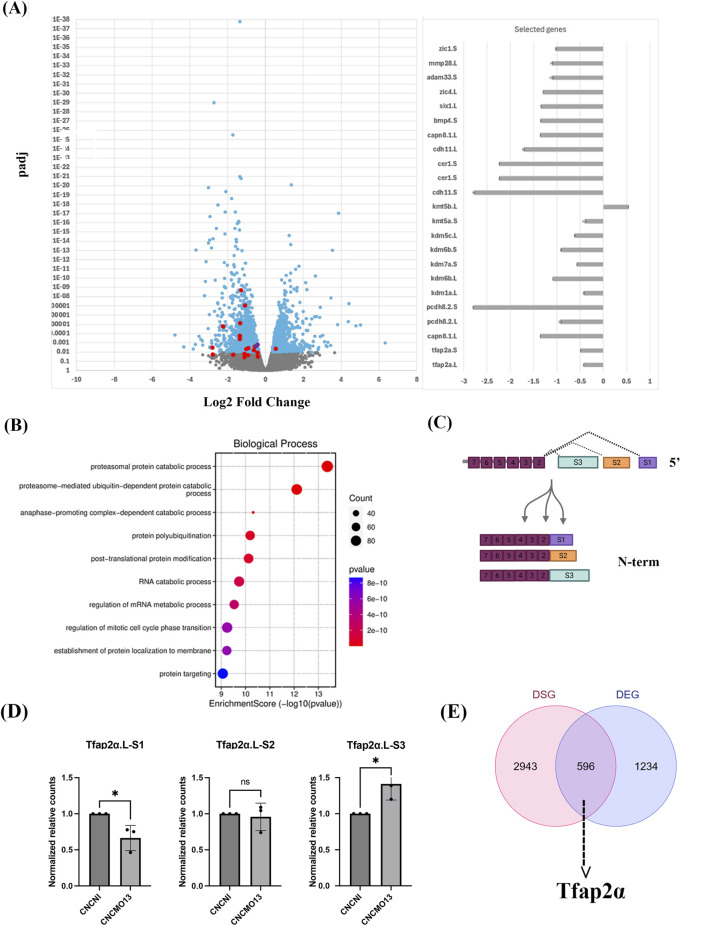
Loss of Adam13 affects gene expression and splicing in the cranial neural crest (CNC). **(A)** Volcano plot showing differential gene expressions in CNC cells dissected from stage 17 embryos from Adam13 knockdown (MO13) versus wild-type (WT) embryos. Log_2_(fold change) is plotted on the *x* axis, with negative values indicating downregulation and positive values indicating upregulation. The significantly differentially expressed genes (DEGs; adjusted *p* < 0.01) are highlighted in blue, and selected genes of interest are marked in red (adjusted *p* < 0.05). The identity and fold changes of these genes are provided in the bar graph on the right; *Tfap2α*-L and S are represented by two purple dots. The complete list of genes identified is provided in Supplementary Table S1. **(B)** Gene set enrichment analysis (GSEA) of the biological processes affected by the DEGs in Adam13 protein knockdown CNC cells. The dot size corresponds to the number of genes in the pathway, while the dot color represents the *p*-value. The enriched biological processes are related to protein and RNA modifications. **(C)** Schematic representation of the *Tfap2α* gene showing the three alternate transcription start sites (TSSs; S1–S3) that result in different protein isoforms with distinct N-terminal sequences. **(D)** Bar graph depicting the relative expressions of *Tfap2α* variants at the S1, S2, and S3 TSSs in response to Adam13 knockdown in the CNC cells. **(E)** Venn diagram showing the overlap between differentially spliced genes (DSGs) and DEGs, with *Tfap2α* serving as an example gene with both significant (adjusted *p* < 0.01) splicing and expression changes.

We previously demonstrated that loss of *Adam13* in the CNC cells causes widespread changes in gene expression, as determined by microarray analysis (Cousin et al., 2011). To further explore these changes at greater resolution, we repeated the experiments by dissecting 20 CNC explants from either control embryos or embryos injected with 10 ng of *Adam13* morpholino (MO13) to prevent translation of more than 90% of the Adam13 protein (Cousin et al., 2011). We used embryos from three different batches (different males and females to obtain biological replicates) at stage 17 to extract the mRNA for bulk RNA sequencing (RNAseq; Supplementary Table 1). The RNAseq data confirm that most of the genes identified in our earlier microarray study are also significantly dysregulated in the present dataset at an adjusted *p*-value of 0.05 (Figure 1A). Some of these overlapping genes are highlighted in red in the volcano plot and further quantified in the adjacent bar graph (right). Note that the line of significance in the volcano plot is set at 0.01 and not 0.05 to highlight the most significant genes, while the original microarray analysis was conducted at *p* < 0.05. The dysregulated genes include direct transcriptional targets of *Adam13* (such as *tfap2α*) as well as indirect targets (such as *calpain8* and the protocadherin *pcdh8.2*). Additionally, we observed altered expressions of genes known to be critical for CNC development, including *cadherin*-*11*, *mmp28*, *zic1*, and *bmp4*, along with genes essential for general embryonic development, such as *six1* and *cerberus1* (bar graph in Figure 1A). Gene ontology analysis revealed that the loss of *Adam13* significantly affected biological processes related to RNA processing, splicing, and protein modifications (Figure 1B). This suggests that Adam13 may influence both gene expression and protein stability in the neural crest cells.

### *Tfap2α* regulation by Adam13 and transcriptional variants

Following this global analysis, we focused specifically on *tfap2α* as a direct transcriptional target of *Adam13*. RNAseq analysis confirmed that *tfap2α* expression is significantly reduced in CNC cells lacking Adam13 for both the L and S alloalleles (purple dots in Figure 1A). Similar to humans, *tfap2α* is transcribed into multiple isoforms in *Xenopus*, including via alternative transcription start sites (TSSs), producing protein variants with distinct N-terminal sequences (Figure 1C). We identified three transcription start sites (S1, S2, and S3) and named them according to their positions relative to the 5′ end of the gene. Two of these isoforms were previously shown to be differentially expressed in cancer and possess distinct transcriptional activities (Berlato et al., 2011). We first calculated the relative expression of each variant in wild-type embryos by mapping the sequences linking each alternate exon 1 to the common exon 2. All three variants are expressed at similar levels in the CNC given the wide variation between outbred embryos (average S1 = 1; S2 = 0.9; S3 = 0.8; no statistical differences). We next compared the relative expressions of these variants in control and knockdown embryos (Figure 1D). In Adam13-deficient CNCs, the S1 isoform is significantly downregulated, while the S2 isoform remains unchanged and S3 is upregulated.

These results suggest that Adam13 promotes expression of the S1 isoform in the CNC to encode the shortest N-terminal variant while repressing usage of the S3 start site. Given the possible functional differences between these isoforms, this shift in isoform utilization may have important consequences for CNC development and GRNs.

### Adam13 impacts splicing and isoform selection broadly

To determine whether *tfap2α* was uniquely regulated at the isoform level, we analyzed the global changes in RNA splicing upon *Adam13* loss (Figure 1E). We identified 3,539 genes with significant differences in splicing (adjusted *p* < 0.01), many of which overlap with the differentially expressed genes (DEGs; 596 of 1,830). This shows that loss of Adam13 impacts splicing of a large number of genes in the neural crest cells.

For the remainder of the study, we focused on two genes, namely, *tfap2α* (direct target of Adam13) and *calpain8* (secondary target regulated by *Tfap2α* that is essential for CNC migration and known to partially rescue *Adam13* deficiency) (Cousin et al., 2011; Khedgikar et al., 2017), to understand how Adam13 regulates gene expression.

### Histone methylation changes in CNC cells lacking Adam13

*Adam13* interacts with the transcription factor Arid3a in the CNC cells to promote *tfap2α* expression (Khedgikar et al., 2017). Although Arid3a has a well-characterized role in mesoderm induction in *Xenopus* by regulating *Brachyury* downstream of TGFβ/Smad signaling (Callery et al., 2005), this pathway does not appear to mediate Arid3a activity in the CNC cells (Khedgikar et al., 2017). In other systems, ARID3A/ARID3B have been shown to regulate gene expressions by interacting with histone demethylases, such as KDM4C, to reduce H3K9 trimethylation and thereby promote chromatin accessibility. This has been observed both in human cancers and during nephric tubule regeneration in *Xenopus* (Liao et al., 2016; Suzuki et al., 2019).

**FIGURE 2:**
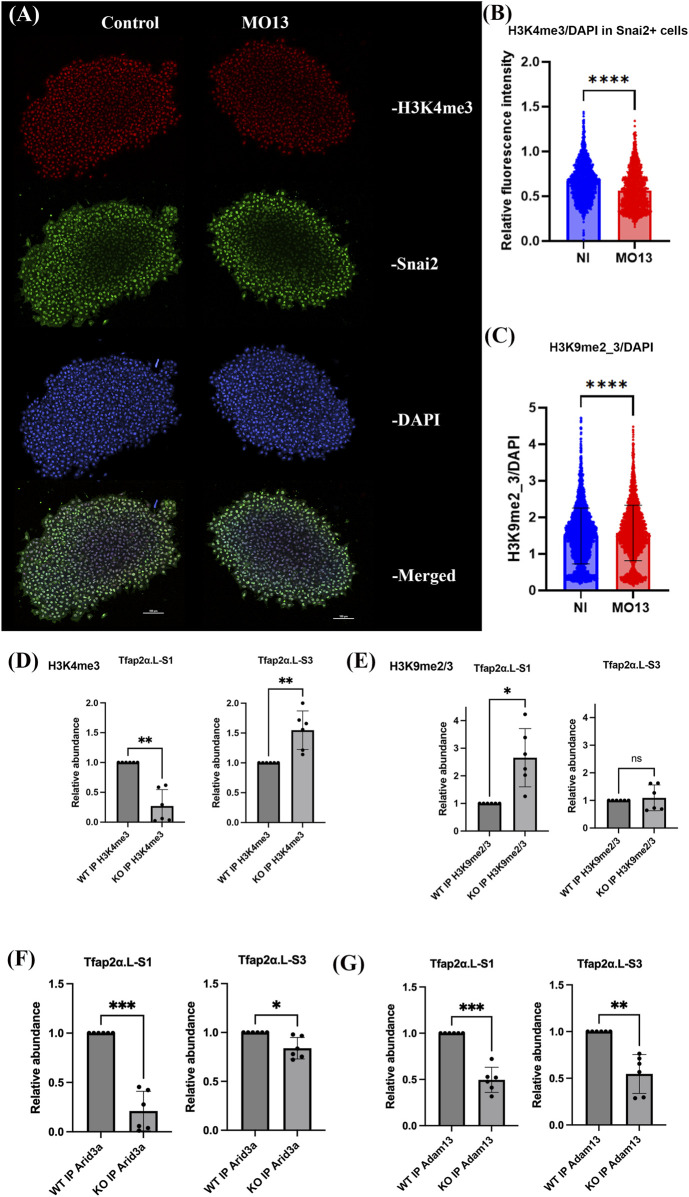
Loss of Adam13 leads to changes in histone H3 methylation. **(A)** Immunofluorescence images showing H3K4me3 (red), Snai2 (green), and DAPI (blue) staining of the CNC explants from control and Adam13 knockdown (MO13) embryos. The maximum projection images from confocal microscopy are shown here. **(B)** Bar graph quantifying the H3K4me3 fluorescence intensities normalized to DAPI. Snai2 staining confirms the identity of the CNC. The data represent at least nine explants (three independent experiments with 100+ cells per explant). Statistical significance is indicated as ****p < 0.0001. **(C)** Bar graph quantifying H3K9me2_3 fluorescence intensities normalized to DAPI. The statistical analysis was the same as in **(B)**. **(D–G)** Chromatin immunoprecipitation (ChIP) from *Xenopus* embryos at the neurula stage 20. The bar graphs represent the relative abundances of chromatin associated with different proteins by ChIP-qPCR analysis: **(D)** H3K4, **(E)** H3K9, **(F)** Arid3a, and **(G)** Adam13. The results were normalized to WT embryos and compared to homozygote knockout embryos for *Adam13*.*L* (KO). Chromatin was immunoprecipitated using antibodies against H3K4me3, H3K9me2/3, Arid3a, and Adam13, as indicated on the *x* axis. The associated chromatin was amplified using primers upstream of the S1 and S3 transcription start sequences of *Tfap2α*.*L*. Six replicates were used for the statistical analyses, and statistical significance is indicated as *p < 0.05, **p < 0.01, ***p < 0.001, and ****p < 0.0001.

Given that Adam13 signaling does not involve the TGFβ pathway in the CNC cells, we tested whether its loss would affect the histone methylation states. We quantified the histone methylation marks in dissected CNC explants from control (wild-type) and Adam13-depleted (MO13-injected) embryos using immunofluorescence staining (Figures 2A,B). The explants were dissected at stage 17 and allowed to migrate on glass bottom dishes coated with fibronectin for 2 h, as described previously (Alfandari et al., 2003). For each quantification, we used three individual explants for each condition in three independent experiments performed on separate days using different adults (total of nine CNC explants for each condition). Counterstaining using antibodies to Snai2 (Horr et al., 2023) (Figure 2A) confirmed that nearly all cells are of neural crest origin. For each explant, we measured the fluorescence of individual nuclei via DAPI staining. In CNC cells lacking Adam13, we observed a significant decrease in H3K4 methylation (an activating chromatin mark; Figures 2A,B) and a corresponding increase in H3K9 methylation (a repressive mark; Figure 2C; Supplementary Figure S1). Although these global changes are small (15% for H3K4 and 5.6% for H3K9), they are statistically highly significant given the large number of measures. These data suggest that Adam13 promotes a more open chromatin state in CNC cells.

### Adam13 modulates chromatin state at alternate TSSs of *Tfap2α*

To test whether these methylation changes occurred specifically at the *tfap2α* promoter, we performed chromatin immunoprecipitation (ChIP) assays using antibodies against H3K4 and H3K9 methylation; here, we focused on the S1 and S3 TSSs using primers designed around these regions (Figure 1D). We performed experiments in both wild-type and homozygote *Adam13* knockout embryos at stage 20. The knockout line (F3 homozygote) harbors a deletion in the first exon of *adam13.L*, which results in a frameshift and complete loss of Adam13 protein in the homozygote adults and embryos (Supplementary Figure S2). The adults are viable, can reproduce normally, and appear normal overall with smaller juvenile average sizes; the adult skeleton is currently being analyzed in detail by our research group.

The ChIP results show that the absence of *Adam13* significantly reduces H3K4 methylation upstream of the S1 start site while increasing H3K4 methylation upstream of S3 (Figure 2D); conversely, H3K9 methylation is increased upstream of S1 but unchanged at S3 (Figure 2E). These findings are consistent with the RNAseq data showing that S1 expression is downregulated and S3 is upregulated in *Adam13*-deficient CNC cells (Figure 1D). To determine whether Arid3a recruitment to these sequences depends on *Adam13*, we repeated the ChIP assays using an antibody against Arid3a (Figure 2F); accordingly, we found that Arid3a binding upstream of S1 requires *Adam13*, whereas binding upstream of S3 occurs independently of *Adam13*. Finally, we tested whether *Adam13* itself is present at the *tfap2α* promoter. Using the *Adam13* knockout embryos as a negative control, we performed ChIP with an *Adam13* antibody to the cytoplasmic domain (Cousin et al., 2011) and found that *Adam13* is associated with both the S1 and S3 transcription start regions of *tfap2α* (Figure 2G). This supports the model that *Adam13* acts directly at the *tfap2α* locus to regulate transcription start utilization; it also suggests that *Adam13* acts as both an activator (S1) and a repressor (S3), as shown by the RNAseq results (Figure 1A).

### Adam13 and Arid3a regulate *tfap2α* expression *in vitro*

**FIGURE 3:**
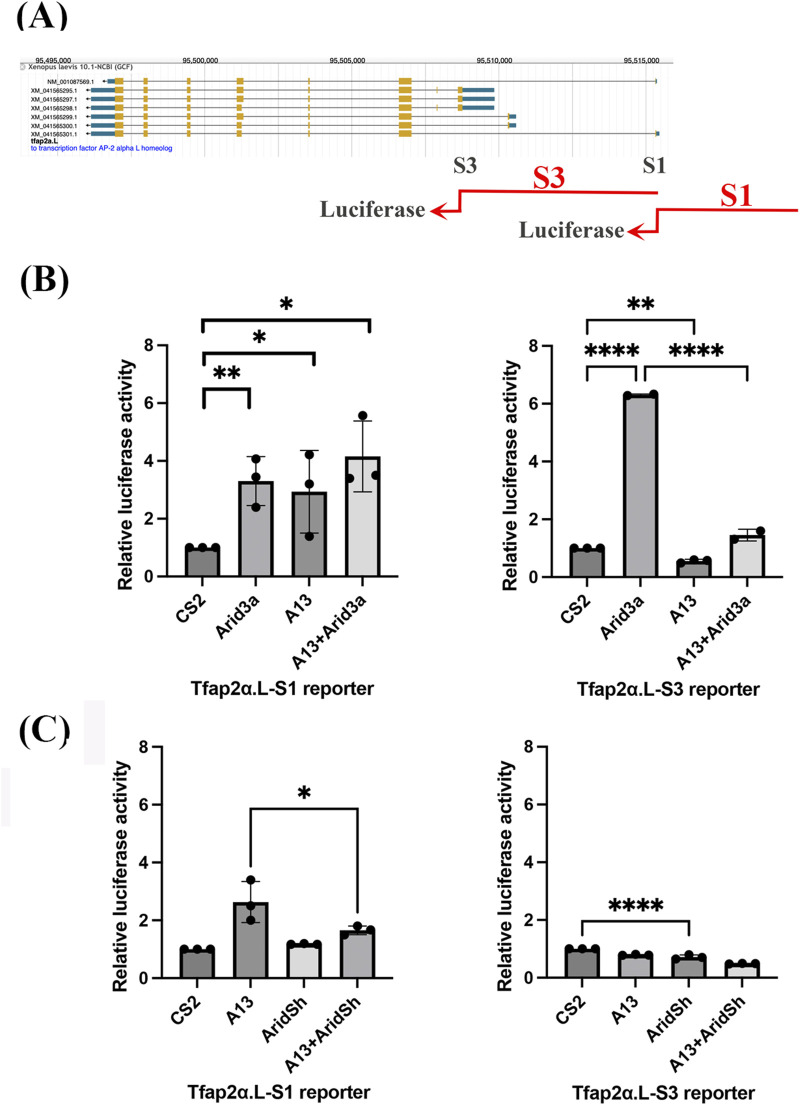
Luciferase assay using *Tfap2α* reporter upstream of the S1 and S3 transcription start sites. **(A)** Schematic representation of the *Xenopus laevis Tfap2α*.*L* gene and S1/S3 transcription start sites used for luciferase reporter constructs. The sequences used to produce the luciferase reporter (red) are aligned to the genome. **(B, C)** Bar graphs showing the relative luciferase activities of S1 and S3 reporter constructs transfected into HEK293T cells together with a CMV Renilla construct with or without *Xenopus* Adam13 and Arid3a. **(B)** Three biological replicates were used for each condition, and protein expression was confirmed by Western blotting. Statistical significance was determined by one-way ANOVA (*p < 0.05, **p < 0.01, and ****p < 0.0001). **(C)** Bar graph showing relative luciferase activities of the S1 and S3 reporters in the presence of shRNA targeting human Arid3a. The data are normalized to the CMV Renilla reporter. Statistical significance was determined by one-way ANOVA (*p < 0.05, **p < 0.01, and ****p < 0.0001).

Opening the chromatin is the first step in allowing transcription factors to bind to the promoter. To determine whether Adam13 and Arid3a are sufficient for regulating *tfap2α* expression, we generated luciferase reporter constructs containing the genomic DNA fragments upstream of the S1 (2,368 bp) or S3 (3,577 bp) transcription starts (Figure 3A). These constructs were transfected into human HEK293T cells either alone or in combination with expression plasmids for Arid3a and/or Adam13. The empty vector (plasmid CS2) was used as a control, and CS2-Renilla was used for normalization. The luciferase assays revealed that both Arid3a and Adam13 independently activated the S1 upstream sequence (Figure 3B). Arid3a alone also activated the S3 reporter construct; however, Adam13 repressed this activation, consistent with the *in vivo* RNAseq data showing reduced S3 usage in the presence of Adam13 (Figure 1D). To assess the contribution of endogenous human ARID3A in this system, we transfected a plasmid encoding human ARID3A shRNA along with the S1 or S3 reporter construct (Figure 3C); knockdown of endogenous ARID3A significantly reduced the S1 reporter activity induced by Adam13 and also reduced the S3 reporter activity. Notably, ARID3A knockdown enhanced *Adam13*-mediated repression of the S3 reporter. These results indicate that endogenous ARID3A contributes to activation of both S1 and S3 reporters in human HEK293T cells and that Adam13 is required for full activation of S1.

### Functional roles of *tfap2α* isoforms in CNC migration

**FIGURE 4:**
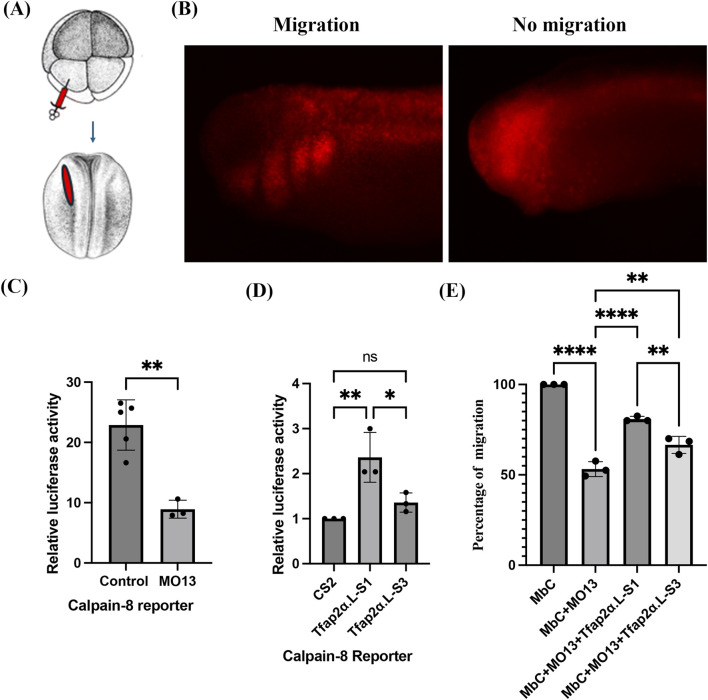
Biological activities of the S1 and S3 *Tfap2α* isoforms. **(A)** Schematic representation of the experimental setup. Top view of an embryo at the 8-cell stage with its dorsal side down (lighter pigment). The injection targets the dorsal animal cells, and these cells produce the red-labeled tissue at the neurula stage (dorsal view stage 18) corresponding to the dorsal anterior quadrant of the embryo that includes the CNC (Moody, 1987). Plasmids, morpholino, and mRNA including lineage tracers can be injected. **(B)** Lateral view with anterior to the left and dorsal side up for representative embryos at stage 24 showing typical scoring. The embryo to the left is a typical sample injected with red fluorescence protein mRNA showing clear CNC migration on the ventral side in all available segments. The embryo to the right is typical of a sample injected with the Adam13 morpholino, in which no migrating neural crest is seen on the ventral side despite correct targeting of the dorsal anterior quadrant. Only embryos identical to the example on the right side are scored as no migration. Embryos with partial migration in any of the posterior segments are scored as migration to obtain a simple binary data point. **(C)** Bar graph representing the luciferase activity of the calpain8 reporter injected as in **(A)**. The reporter activity decreased by approximately 50% when injected in embryos lacking Adam13 (MO13). Embryos selected at the neurula stage based on expression of the lineage tracer in the dorsal anterior quadrant as in **(A)** were tested for luciferase activity and normalized to Renilla. Three to five biological replicates are plotted. Statistical analysis was performed using a t-test with **p < 0.01. **(D)** Bar graph showing the relative calpain8 luciferase reporter expressions in HEK293T cells transfected with the empty vector (CS2), *Tfap2α-L*-S1, or *Tfap2α-L*-S3 constructs. Statistical analysis was performed by one-way ANOVA from three biological repeats (*p < 0.05 and **p < 0.01; ns, *p* > 0.05). **(E)** Bar graph depicting the percentage of embryos with CNC migration in the three biological repeats based on images shown in **(B)**. The number of embryos is as follows: MbC (64), MO13 (53), MO13 + *Tfap2α*-S1 (60), MO13 + *Tfap2α*-S3 (56). Statistical analysis was performed using ANOVA with **p < 0.01 and ****p < 0.0001.

Given that *Adam13* promotes *tfap2α* expression from the S1 start and represses S3 usage (Figure 1D), we investigated the functional significances of these isoforms in CNC development. We first examined whether the S1 and S3 isoforms differed in their abilities to activate *calpain8*, a critical downstream target of *Tfap2α* involved in CNC migration (Cousin et al., 2011). Thus, we constructed a *calpain8* luciferase reporter containing 2,483 bp upstream of the first ATG and injected it into one CNC precursor cell at the eight-cell stage (Figure 4A), along with red fluorescence protein (Figure 4B) as a lineage tracer and CMV Renilla for normalization (Figures 4A,D). Embryos showing strong red fluorescence in the dorsal anterior tissues (properly targeted) were selected for luciferase analysis (both examples in Figure 4B). The wild-type embryos exhibited robust *calpain8* reporter activities, while embryos injected with MO13 showed ∼50% reduction in luciferase signals (Figure 4C), consistent with the RNAseq results (Figure 1A). Upon confirming the activity of the luciferase reporter *in vivo*, we transfected the reporter in HEK293T cells with either of the *Tfap2α* variants. Expression of *tfap2α*-S1 but not *tfap2α*-S3 restored the activity of the *calpain8* reporter (Figure 4D), suggesting that the S1 isoform is the transcriptionally active variant required for *calpain8* expression. We next evaluated whether these isoforms could rescue CNC migration in embryos lacking *Adam13*. Membrane-targeted Cherry (MbC) was injected at the eight-cell stage to trace the CNC cells, as shown in Figure 4A. Injection of MO13 blocked CNC migration in ∼50% of the embryos (Figure 4E), as observed previously (Cousin et al., 2012; Khedgikar et al., 2017); co-injection of *tfap2α*-S1 significantly rescued migration while *tfap2α*-S3 enabled only partial rescue (Figure 4E, *p* < 0.01). The incomplete rescue reflects the fact that *Adam13* plays a critical proteolytic role in cleaving cadherin-11, which is not compensated by *Tfap2α* (McCusker et al., 2009).

### Adam13 is associated with histone methyltransferases

**FIGURE 5:**
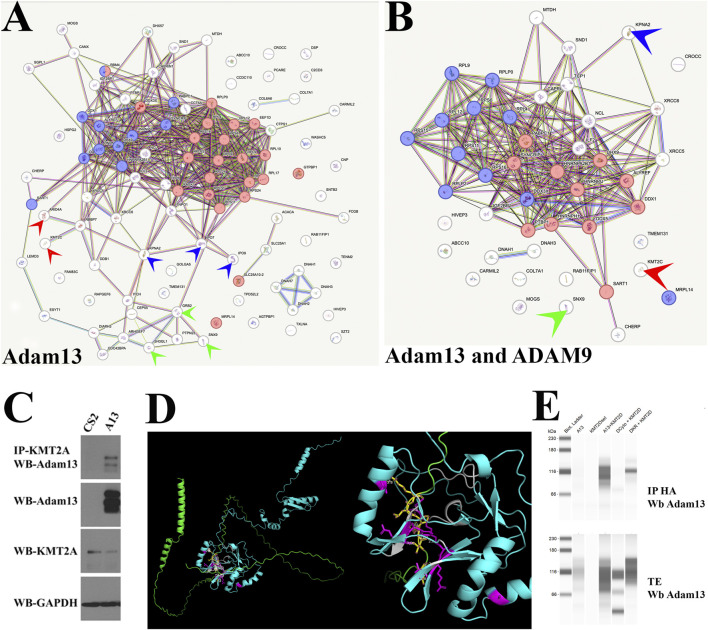
Adam13 binds to multiple nuclear proteins, including histone methyltransferases. Proteomic analyses of proteins **(A)** associated with the cytoplasmic domain of *Xenopus* Adam13 and **(B)** common to *Xenopus* Adam13 and human ADAM9. The protein interactions were analyzed using STRING. Proteins involved in splicing are shown in blue, translation regulation in red, and key interactors are highlighted with colored arrowheads (green, SH3-domain proteins; blue, importins; red, Arid4a and KMT2C). **(C)** Co-immunoprecipitation of Adam13 and endogenous human KMT2A. HEK293T cells were transfected with either the empty vector (CS2) or Adam13 (A13), and the extract was immunoprecipitated using an anti-KMT2A antibody and blotted (WB) with an anti-Adam13 monoclonal antibody (mAb 7C9). The total extracts were blotted with antibodies to Adam13, KMT2A, and GAPDH. **(D)** AlphaFold modeling of the interaction between the cytoplasmic domain of Adam13 (green) and *Xenopus* KMT2D SET domain (cyan). Potential interaction sites are highlighted in yellow (Adam13 amino acids) and purple (KMT2D amino acids). The image on the right side is a magnified version in the same orientation. **(E)** Capillary Western blotting showing co-immunoprecipitation of HA-tagged KMT2D SET domain and Adam13 in HEK293T cells. The upper panel shows co-immunoprecipitation with HA blotted with Adam13 (mAb 7C9), while the lower panel shows the total extract (TE) for Adam13 detection. The Adam13 mutant lacking its cytoplasmic domain (ΔCyto) does not bind KMT2D, while the mutant missing the nuclear localization signal delta KR binds KMT2D. The mAb 7C9 binds to the extracellular domain of Adam13 present in all Adam13 constructs.

Given that Adam13 binds to the *tfap2α* promoter and promotes H3K4 methylation at S1, we sought to identify the interacting proteins that might mediate this effect. Using mass spectrometry, we analyzed the protein complexes associated with full-length Adam13 and a version lacking its cytoplasmic domain (ΔCyto). In addition, we generated a BioID construct by fusing BirA to the cytoplasmic domain of Adam13 to enable proximity labeling (with biotin) of the transiently interacting proteins (Figure 5A). The proteins were considered specific if they were detected in both the immunoprecipitated and biotinylated samples but absent from the ΔCyto controls. Using stringent criteria (≥2 unique peptides and 95% confidence), we identified 120 interacting proteins (Figure 5A; Supplementary Table S2). These included known cytoplasmic interactors such as SH3-domain-containing proteins (e.g., Grb2, Snx9, and Sh3Gl1; green arrowheads in Figure 5A), novel proteins such as nuclear importins (IPO7, IPO9, and KPNA2; blue arrowheads in Figure 5A), and chromatin modifiers such as KMT2C and Arid4A (red arrowheads in Figure 5A). Pathway enrichment analysis revealed that the interacting proteins are enriched in functions related to RNA splicing (blue circles in Figure 5A) and translation (red circles in Figure 5A). To confirm that these interactions were not limited to Adam13 in *Xenopus*, we performed similar experiments with endogenous human ADAM9 and identified 55 overlapping proteins (Figure 5B), including importins (blue arrowhead), SH3-domain-containing proteins (green arrowhead), KMT2C (red arrowhead), and other chromatin regulators (Supplementary Table S3). Given that the KMT2 family members catalyze H3K4 methylation, we also focused on validating these interactions. In HEK293T cells, we confirmed that Adam13 coprecipitates with endogenous KMT2A (Figure 5C). Using AlphaFold3 predictions, we identified a potential interaction site between Adam13 and the SET domain of KMT2 (Figure 5D). In *Xenopus*, KMT2D is expressed in the CNC and is essential for CNC migration (Schwenty-Lara et al., 2020). Co-immunoprecipitation assays using the SET domain of KMT2D in *Xenopus* confirmed direct interaction with Adam13, which was absent in the ΔCyto mutant (Figure 5E).

These results strongly support a model in which *Adam13* interacts with KMT2 histone methyltransferases to promote H3K4 methylation at specific promoters, such as *tfap2α*-S1, thereby regulating transcriptional programs essential for CNC development.

## Discussion

### ADAM proteins in the nucleus

The roles of the proteolytic activities of ADAM proteins are well documented and essential for all the developmental process studied so far (Alfandari and Taneyhill, 2018; Blobel, 2005; Dreymueller et al., 2017; Lambrecht et al., 2018; Tousseyn et al., 2006), but the presence and functions of ADAM in the nucleus are less documented and unclear. Recent studies have identified three ADAM proteins (Adam9, Adam10, and Adam13) localized in the nucleus, where their roles are still being elucidated (Cousin et al., 2011; Lin et al., 2021; Tousseyn et al., 2009). Although the nuclear function of Adam10 remains underexplored, Adam9 has been shown to act as a repressor of genes inhibiting angiogenesis, with its promoter-bound activity resulting in the expression of pro-angiogenesis genes in cancer cells (Lin et al., 2021). In *Xenopus*, we demonstrated that the cytoplasmic domain of Adam13 undergoes γ-secretase-mediated cleavage and translocates to the nucleus, where it interacts with the transcription factor Arid3a to activate the expression of genes critical for CNC migration, including *Tfap2α* (Cousin et al., 2011; Khedgikar et al., 2017).

Here, we report that loss of Adam13 leads to changes in histone modifications, with decreased trimethylation of H3K4 and increased trimethylation of H3K9, suggesting that Adam13 may promote a more transcriptionally permissive chromatin state (Figure 2). Our mass spectrometry data show that several interacting proteins are involved in the histone modifications, including the methyltransferase KMT2A that adds methyl groups to H3K4 and the KDM family of demethylases that remove methyl groups from H3K9 (Figure 5). These findings suggest that Adam13 may regulate transcriptional activation through a chromatin-modifying complex involving Arid3a and histone-modifying enzymes.

By focusing on the *Tfap2α* locus, we found that loss of Adam13 reduces H3K4 methylation and increases H3K9 methylation at the upstream region of the promoter, which are correlated with decreased Arid3a binding and reduced *Tfap2α* transcription (Figure 2). Our luciferase reporter assays suggest that Adam13 and Arid3a can independently regulate transcription when chromatin is accessible, supporting the idea that histone modifications may precede transcriptional regulation by this complex. Interestingly, the results from the third TSS of *Tfap2α* show that Adam13 acts as both an activator and a repressor depending on the genomic context, consistent with our observation that similar numbers of genes are upregulated and downregulated in CNC cells lacking Adam13 (Figure 1). These findings suggest that Adam13 may play a context-dependent role in modulating gene expression through chromatin remodeling.

### Splicing regulation by Adam13

Our study reveals that Adam13 also regulates alternative splicing in the CNC. In the absence of Adam13, we found that 3,539 genes show differential splicing and that this number is significantly higher than the 1,830 differentially expressed genes (DEGs). This suggests that Adam13 impacts splicing at multiple levels. For instance, changes in histone modifications at the alternative TSSs, such as those observed in *Tfap2α*, likely influence exon usage as recent studies have shown that local histone modifications can affect splicing patterns (Zhou et al., 2014). Furthermore, our mass spectrometry results show that both Adam9 and Adam13 interact with splicing-associated proteins, including RNA helicases, PTBP1, and HNRNPs, suggesting that ADAMs may be a part of multiprotein complexes involved in RNA trafficking, splicing, and translation regulation (Figure 5).

### Biological relevance of *Tfap2α* isoforms

Differential regulation of the *Tfap2α* isoforms by Adam13 is particularly notable in this work. We observed that Adam13 promotes expression of the S1 isoform from the most upstream TSS (S1) while repressing the S3 isoform. These isoforms differ in the first 52 amino acids that are not associated with an obvious functional domain, yet the biological activities of the isoforms are distinct. The S1 isoform is more efficient at rescuing CNC migration in the absence of Adam13, likely owing to its ability to activate *calpain8* expression, which is not possible with the S3 isoform (Figure 4). Interestingly, similar isoform-specific differences in *Tfap2α* regulation were observed in human breast cancer, where the two isoforms of *Tfap2α* also differed in their N-terminal sequences. The S3 isoform (but not the S1 isoform) contains a SUMOylation site that is required for its repressive activity on cyclin-D3 gene expression (Berlato et al., 2011). Given the widespread expression of *Tfap2α*, it is likely that the different functions of *Tfap2α* in specific tissues are driven by the variant expressed rather than the overall level of mRNA expressed. Identifying the partners and transcription targets of the different isoforms of *Tfap2α* is thus required to understand the complete action mechanisms of the protein.

### Conclusion

Our findings support the hypothesis that Adam13 is part of a large protein complex involved in cell adhesion, cell migration, gene expression, and chromatin remodeling. Rather than simply activating or repressing genes, Adam13 modulates the expressions and splicing of existing genes. We propose that Adam13 may function as a “sensor” at the cell surface by integrating extracellular signals and adaptively regulating gene expressions. The associations of Adam13 with calcium-binding proteins, ion channels, and extracellular matrix components further support this hypothesis, suggesting that Adam13 may respond to ionic fluctuations or other extracellular matrix signals to modulate gene expressions accordingly.

## Materials and methods

### Antibodies

The following antibodies were used in this study: monoclonal antibody to ribophorin-1 (Rpn1) was used as loading control (RRID: AB_2687673); mouse monoclonal antibody to H3K9me2/3 (Cell Signaling Technology; cat. no. 5327, RRID: AB_10695295); mouse monoclonal to Arid3a (DSHB; cat. no. PCRP-ARID3A-1E9, RRID: AB_2618410); rabbit monoclonal to H3K4me3 (Cell Signaling Technology; cat. no. 9751, RRID: AB_2616028); rabbit monoclonal to KMT2α (Cell Signaling Technology; cat. no. 14197, RRID: AB_2688010); mouse monoclonal to TFAP2α (DSHB; cat. no. 3B5, RRID: AB_2202275); mouse monoclonal to anti-flag tag (DSHB; cat. no. 12C6c, RRID: AB_2890618); and rabbit polyclonal against Sox9 (Sigma; AB5535). Anti-Adam13 antibodies against the cytoplasmic domain and extracellular domains are described elsewhere (Cousin et al., 2011; Horr et al., 2023). The snai2/slug antibody (DA1A8Slug) is as described in Horr et al. (2023).

### Morpholinos and DNA constructs

Morpholino antisense oligonucleotides (Gene Tools, Philomath, OR, United States) were designed to block the translation of Adam13 (MO13; GTC​CCA​GCC​GAC​CCT​CC), and *adam13.L* was cloned in pCS2 vector. The BioID2 tag (Kim et al., 2016) was added to *adam13.L* using Takara infusion cloning at amino acid 893 within the cytoplasmic domain (NSAT-BioID2-QLKG). All Adam13 constructs were performed on the “rescue” construct that has silent mutations at the morpholino binding sites. The BioID constructs were performed on the wild-type *Adam13* as well as E/A proteolytically inactive mutant (A13 E/A) and mutant lacking the cytoplasmic domain (ΔCyto). The *Tfap2α.L*-S1 and *Tfap2α.L*-S3 were cloned from *Xenopus* embryos using Takara infusion cloning. The Arid3a clones used in this work are described elsewhere (Khedgikar et al., 2017). The luciferase reporters were all cloned into pGl3 (Addgene). The genomic sequences for the putative promoters of *Xenopus laevis* were cloned based on their positions in the genome upstream of the TSSs. The *Tfap2α*-S1 construct corresponds to 2,368 bp from the first ATG, while the *Tfap2α*-S3 construct corresponds to 3,577 bp from the third ATG and does not overlap with the S1 region but contains the S2 genomic sequence. The *calpain8*.*L* construct corresponds to 2,483 bp upstream of the first ATG. Human Arid3a shRNA was purchased from Sigma-Aldrich (TRCN0000013791, target sequence: CCC​TAA​GAT​CAA​GAA​AGA​GGA).

### Injections, microdissections, and mRNA synthesis

SP6 polymerase was used for capped mRNAs synthesis after digestion using Not1 (Cousin et al., 2011). The injectors were calibrated using a 1-µL capillary needle (MicroCaps, Drummond, PA, United States); the injection pressure was set at 15 psi, while the injection time was set between 50 and 200 ms to obtain a 5 nL delivery volume. The embryos were injected at the 1-cell, 2-cell, 8-cell, and 16-cell stages, as described previously (Cousin et al., 2012), and grown at 14 °C–15 °C until they could be scored for neural tube closure or CNC cell migration. For each injection, the percentage of fluorescent CNC cells present in the branchial pathway was normalized with that of the control embryos and set to 100%.

### Generation of Adam13 knockout line

The targets in exons 1 and 5 of *adam13.L* were identified using CRISPRscan (https://www.crisprscan.org/) (Moreno-Mateos et al., 2015), and the second nucleotide of each single guide RNA (sgRNA) was modified to a G at the 5′ end for increased mutagenic activity (Gagnon et al., 2014), such that T1: GGG​CAC​ATG​GCT​GGG​ACT​CG and T2: GGA​GGT​GGT​GAG​GGC​GAC​AG. The sgRNAs were synthesized by *in vitro* transcription of the sgRNA polymerase chain reaction (PCR) template using the T7 MEGAscript kit (Ambion, cat. no. AM1334). To generate eggs, inbred *Xla.J-strain* ^*NXR*^ (RRID: NXR_0024) females of *X. laevis* were first given 35 U of pregnant mare serum gonadotropin (PMSG; BioVendor, cat. no. RP17827210000) followed by 350 U of human chorionic gonadotropin (hCG; BioVendor, cat. no. RP17825010) to induce ovulation. The eggs were then fertilized using methods outlined previously in Shaidani et al. (2021). The founders were generated by co-injecting 250 pg each of sgRNAs 1 and 2 with 500 pg of Cas9 protein per embryo at the single-cell stage. The embryos were collected and genotyped using Qiagen DNeasy Blood and Tissue (cat. no./ID: 69506). The genomic DNA was amplified using the following PCR primers: exon 1 forward primer 5′-GTG​TTG​GGT​GAG​TTT​ATT​GGG​C-3′ and reverse primer 5′-TGA​TAG​AGC​CAG​GCT​ATC​AGG​G-3’; exon 5 forward primer 5′-CTT​CGG​ACA​TTG​TGA​TCC​TTG​C-3′ and reverse primer 5′-TTG​ATG​AGA​TAT​AGT​CGC​CCG​G-3’. The embryos were obtained by outcrossing seven F0 *adam13* knockout males with the J-strain females; the F1 embryos were then genotyped to determine the presence of germline mutations. Of the seven males, only three had germline mutations in exon 1 (male 2: 13/+, male 6: 7/+, male 7: 13/+), while no mutations were found in exon 5. The offspring from the second male included both males and females that were then raised through metamorphosis and intercrossed to generate -13/-13 *adam13* knockouts. This mutation results in a premature stop following amino acid 22. The -13/-13 *adam13* mutants (*Xla.adam13* ^*emNXR*^, RRID: NXR_2117) are available from the NXR.

### Cell culture and transfection

HEK293T (ATCC; CRL-3216 RCB2202) cells were cultured in RPMI medium supplemented with 10 U/mL of penicillin/streptomycine, 2 mM of L-glutamine, 0.11 mg/mL of sodium pyruvate, and 10% fetal bovine serum (HyClone, South Logan, UT, United States). The transfections were performed using polyethylenimine (PEI, Polysciences Inc.); for each transfection, 1 µg of DNA was mixed with 10 µg of PEI in 200 µL of RPMI medium (HyClone) at room temperature for 15 min prior to adding to the cells dropwise. The medium was changed 24 h post transfection.

### Immunoprecipitation and Western blots

HEK293T cells, embryos, and CNC explants were extracted in 1× MBS-1% Triton X-100, protease phosphatase inhibitor cocktail 1× (Thermo Fisher Scientific), and 5 mM of EDTA. The immunoprecipitations were performed using 1–5 µg of antibody bound to protein A/G magnetic beads (Thermo Fisher Scientific) and incubated overnight at 4 °C. The beads were washed thrice with extraction buffer for 5 min each at room temperature, before being eluted in 2× reducing Laemmli sample buffer. All proteins were separated in 5%–22% gradient SDS-PAGE gels and transferred to polyvinylidene fluoride membranes (Millipore, Billerica, MA, United States) using a semi-dry transfer apparatus (Hoefer).

### Immunofluorescence

The CNC cells were dissected at stage 17 and placed on fibronectin-coated glass bottom plates (20 µg/mL) for 3 h at 18 °C (Alfandari et al., 2003; Cousin and Alfandari, 2018). The explants were fixed using MEMFA (0.1 M of MOPS at pH 7.4, 2 mM of EGTA at pH 8, 1 mM of MgSO_4_, and 4% paraformaldehyde) for 1 h and permeabilized using 0.5% TX100 in 1× MBS with 100 mM of glycine for 1 h. The explants were then blocked using phosphate-buffered saline (PBS) containing 10% heat-inactivated goat serum, 1% bovine serum albumin, and 0.1% Tween for 1 h prior to incubation in the same buffer with the primary antibodies overnight at 4 °C. The CNC explants were washed in PBS-Tween 0.1% (PTw) thrice for 15 min each and blocked again for 1 h at room temperature using the blocking solution prior to incubation with the secondary antibody and Hoechst 33342 for 1 h at room temperature. The explants were washed thrice in PTw and imaged using a Nikon confocal microscope (A1R HD25).

### Immunofluorescence analysis

The immunofluorescence images were acquired at the same gain and power for all explants in a given experiment, and the images were analyzed using GA3 Nikon. The intensity of the nucleus was measured with a mask using the marker of CNC Sox9 or Snai2, and the intensity of the target was normalized with the intensity of Hoechst 33342. The t-test was used to determine significance from at least three independent experiments.

### Mass spectrometry

The proteins extracted and immunoprecipitated with the target-specific antibodies from embryos or HEK293T cells were resuspended in 8 M of urea and processed with trypsin/lysine-C according to manufacturer instructions (Promega). All samples were cleaned using ZipTip (Fisher Scientific). For the BioID experiments, the HEK293T cells were transfected with Adam13-BioID tagged constructs and grown in a medium depleted for biotin (streptavidin depletion). Biotin was then added to the medium at various time points after transfection, and the transfected cells were either extracted directly as described above or separated by cytoplasmic and nuclear extraction (Pierce NE-PER kit). The biotinylated proteins were then purified using streptavidin magnetic beads (Pierce) before denaturation in 8 M of urea and processing with trypsin/lysine-C according to manufacturer instructions (Promega). Tandem mass spectrometry (MS/MS) was performed using a Thermo Orbitrap Fusion; the mass spectral data were obtained at the University of Massachusetts Mass Spectrometry Core Facility (RRID: SCR_019063). All MS/MS samples were analyzed using Sequest (Thermo Fisher Scientific, San Jose, CA, United States; version IseNode in Proteome Discoverer 2.4.1.15), which was set to search a *Xenopus* protein database produced from the *X. laevis* genome (Xenbase; 72,266 entries) or the human protein database (UniProt Human) containing the sequences for *Xenopus* Adam13, Arid3a, and tfap2α (126,358 entries) by assuming trypsin as the digestion enzyme. Sequest was searched with a fragment ion mass tolerance of 0.60 Da and parent ion tolerance of 10.0 ppm, with carbamidomethyl of cysteine specified as a fixed modification. Oxidation of methionine, acetylation of the N-terminus, as well as phosphorylation of serine, threonine, and tyrosine were specified as the variable modifications in Sequest.

The following were the criteria used for protein identification. Scaffold (version 5.0.1, Proteome Software Inc., Portland, OR, United States) was used to validate the MS/MS-based peptide and protein identifications. The peptide identifications were accepted if they could be established at >95.0% probability by the PeptideProphet algorithm (Keller et al., 2002) with Scaffold delta-mass correction. The protein identifications were accepted if they could be established at >99.0% probability and contained at least two unique identified peptides; the protein probabilities were assigned by the Protein Prophet algorithm (Nesvizhskii et al., 2003). Proteins containing similar peptides that could not be differentiated based on MS/MS analysis alone were grouped to satisfy the principles of parsimony. Proteins sharing significant peptide evidence were grouped into clusters.

### RNA sequencing and analysis

The RNA was prepared using Roche RNA extraction kit (cat. no. 11828665001) from twenty dissected CNCs at stage 17. The isolated RNAs were sent to Azenta Life Sciences for paired-end long-read sequencing (2×150, ∼60 million reads), and the sequenced RNAs were trimmed for adapters and low-quality reads using Trimmomatic (Bolger et al., 2014). The resulting reads were mapped to the *X. laevis* genome v10.1 using Spliced Transcripts Alignment to a Reference (STAR) (Dobin et al., 2013). The mapped reads were counted for genes as well as exon-specific counts using feature counts in the Subread package (Liao et al., 2013). Differences in fold changes between the conditions were analyzed using DESeq2 (Love et al., 2014). For the pathway analysis and heatmap preparation, the gene names were converted into human orthologs. The pathway analysis was performed using the GSEApy library (Fang et al., 2023) and gene set enrichment analysis (GSEA) (Subramanian et al., 2005) with clusterProfiler (Wu et al., 2021); the sashimi plots were obtained using Integrative Genomics Viewer (IGV) (Robinson et al., 2023; Robinson et al., 2011), while custom heatmaps were obtained using geneset from GSEA.

### Statistical analysis

When not specified in the figure legends, two-tail unpaired Student’s t-test was performed for two samples. For more than two samples, we performed one-way ANOVA. All statistical analyses were performed in GraphPad Prism software.

## Data Availability

The datasets presented in this study can be found in online repositories. The names of the repositories and accession numbers can be found in the article/Supplementary Material.
