# Supplementary figures and images for: Adam13 interacts with large protein complexes to regulate histone modification and gene expression
Source: Front Cell Dev Biol. 2026 May 7;14:1824619. doi: 10.3389/fcell.2026.1824619 (PMC13189808; doi:10.3389/fcell.2026.1824619)

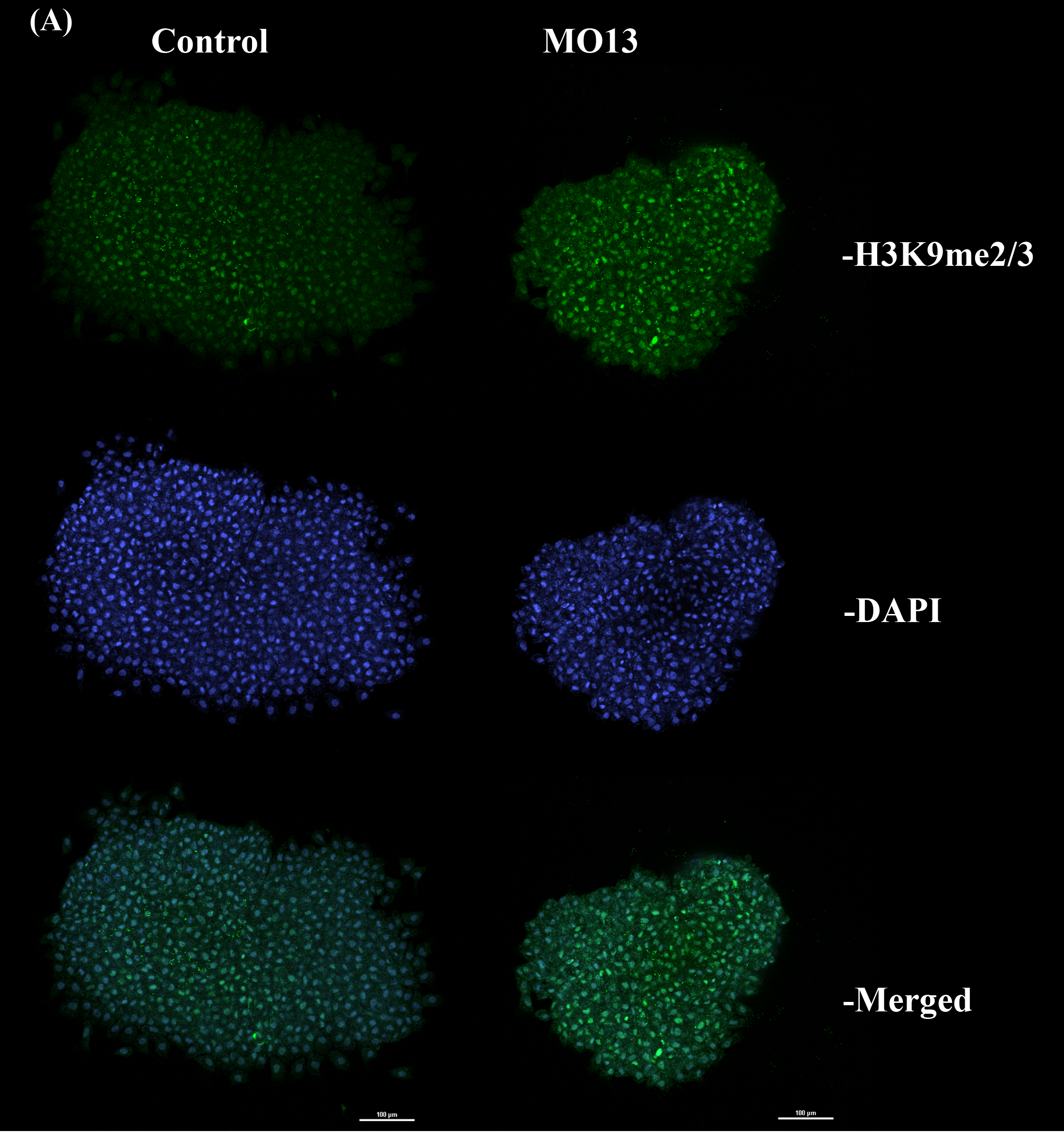

Supplement: Supplementary file 3 [file Image2.tif]

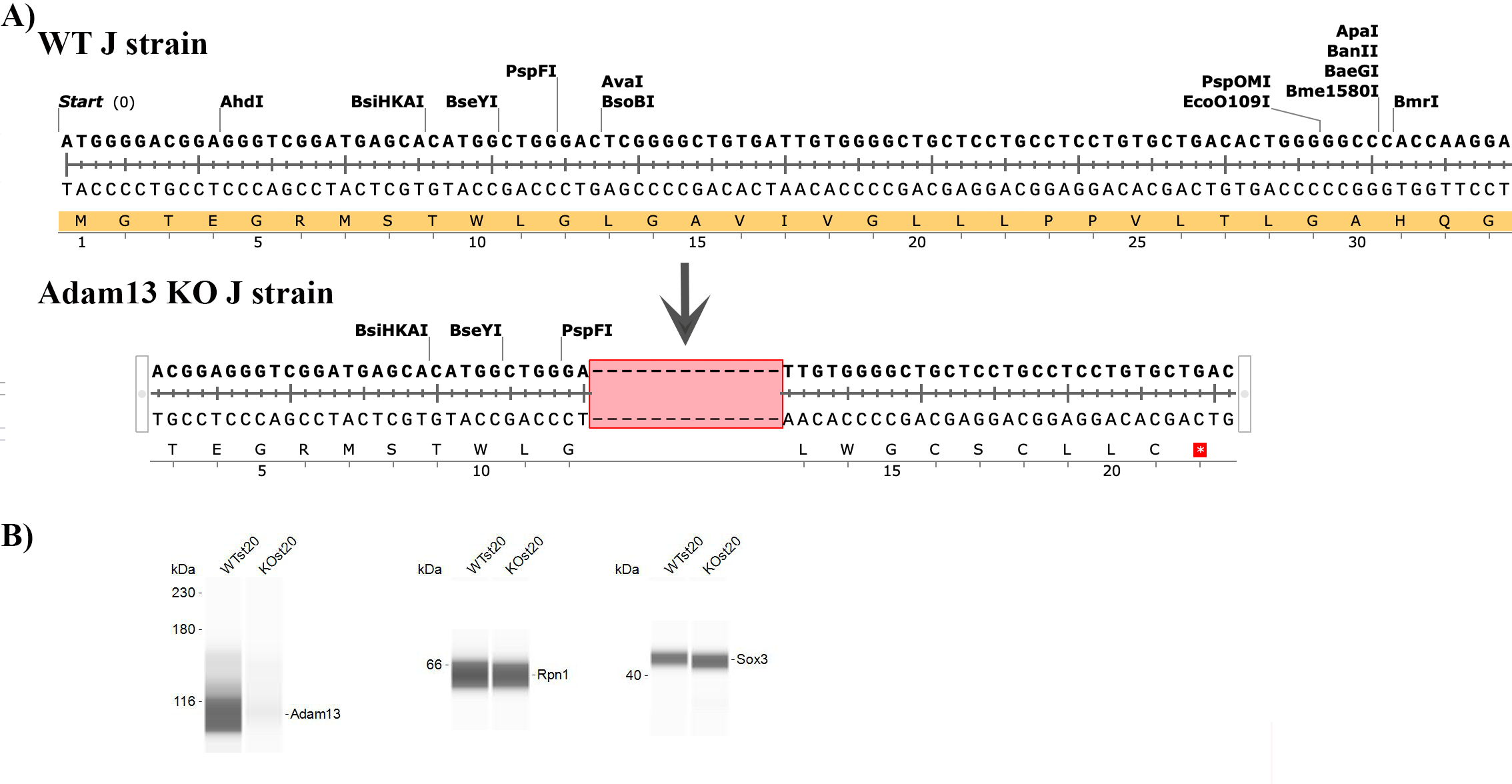

Supplement: Supplementary file 4 [file Image1.tif]
